# Chiral Ionic Liquids: Structural Diversity, Properties and Applications in Selected Separation Techniques

**DOI:** 10.3390/ijms21124253

**Published:** 2020-06-15

**Authors:** Jolanta Flieger, Joanna Feder-Kubis, Małgorzata Tatarczak-Michalewska

**Affiliations:** 1 Department of Analytical Chemistry, Medical University of Lublin, 20-093 Lublin, Poland; malgorzatatatarczakmichalewska@umlub.pl; 2 Department of Process Engineering and Technology of Polymer and Carbon Materials, Faculty of Chemistry, Wrocław University of Science and Technology, 50-370 Wrocław, Poland; joanna.feder-kubis@pwr.edu.pl

**Keywords:** chiral ionic liquids, imidazolium salts, ammonium salts, tunable physicochemical properties, enantioseparation, chromatography, extraction

## Abstract

Ionic liquids (ILs) are chemical compounds composed of ions with melting points below 100 °C exhibiting a design feature. ILs are commonly used as the so-called green solvents, reagents or highly efficient catalysts in varied chemical processes. The huge application potential of ionic liquids (IL) justifies the growing interest in these compounds. In the last decade, increasing attention has been devoted to the development of new methods in the synthesis of stable chiral ionic liquids (CILs) and their application in various separation techniques. The beginnings of the successful use of CILs to separate enantiomers date back to the 1990 s. Most chiral ILs are based on chiral cations or chiral anions. There is also a limited number of CILs possessing both a chiral cation and a chiral anion. Due to the high molecular diversity of both ions, of which at least one has a chiral center, we have the possibility to design a large variety of optically active structures, thus expanding the range of CIL applications. Research utilizing chiral ionic liquids only recently has become more popular. However, it is the area that still has great potential for future development. This review aimed to describe the diversity of structures, properties and examples of applications of chiral ionic liquids as new chiral solid materials and chiral components of the anisotropic environment, providing chiral recognition of enantiomeric analytes, which is useful in liquid chromatography, countercurrent chromatography and other various CIL-based extraction techniques including aqueous biphasic (ABS) extraction systems, solid–liquid two-phase systems, liquid–liquid extraction systems with hydrophilic CILs, liquid–liquid extraction systems with hydrophobic CILs, solid-phase extraction and induced-precipitation techniques developed in the recent years. The growing demand for pure enantiomers in the pharmaceutical and food industries sparks further development in the field of extraction and separation systems modified with CILs highlighting them as affordable and environmentally friendly both chiral selectors and solvents.

## 1. Introduction

The origin of research on ionic liquids dates back to the 19th century when the first information about obtaining salts with characteristic properties of nowadays known as “ionic liquids” appeared in 1888 [1] and 1914 [2]. The first published report on the synthesis of ethyl ammonium nitrate, identified as the first stable room-temperature ionic liquid, was published by Walden in 1914 [2]. Initially, they were defined as “low-melting organic salts” or “molten salts”. However, due to the lack of knowledge on potential applications, this newly discovered category of compounds did not arouse the enthusiasm of the researchers.

This state of affairs changed only at the turn of the twentieth and twenty-first century, when Professor Kenneth R. Seddon, at the conference in Zürich [3], giving a lecture “Molten salt—chemistry and technology (Molten Salt Chemistry and Technology)”, drew the attention of researchers to the potential of these compounds associated with the possibility of designing their numerous functions, including, for example, their role as a substitute for solvents. That conference speech was the follow-up of previous research concluded in 1996 with the publication, in which the term *ionic liquid* was used for the first time [4]. This triggered global research on ionic liquids whose unquestioned leader was Professor Kenneth R. Seddon, mentioned above. Another scientific study that appeared at the parallel time—namely in 1992 and that had a huge impact on the development of research on ionic liquids—was the publication of Wilkes and Zaworotko [5] who described the synthesis of air and water stable low melting imidazolium-based ionic liquid. The research work on these chemical compounds was also carried out by other teams of researchers, whose contributions involved, among others, designing and obtaining chiral ionic liquids [6,7].

Over the years, the interest in ILs has substantially grown and the promising results have encouraged scientists to continue research on a wider scale. In the 21st century, the implementation of research projects related to the design of the properties and structure of new ionic liquids along with the synthesis process, the experimental verification of their functions and the experimental verification of their applications, including those having technological character [8], has become a global trend.

Nowadays, ionic liquids are defined as the chemical compounds composed of a cation and an anion, characterized by a melting temperature below 100 °C [9,10] exhibiting a design feature. Ionic liquid is structurally composed of a cation and anion and each of these ions opens up the possibility of introducing a unique property or function into the molecule. The ionic structure of salts affects their physicochemical properties [11,12], which can be concretized, inter alia, in their non-volatile character, non-flammability, according to the criteria of the Globally Harmonized System [13], thermal stability (even up to 300 °C) [14,15], chemical stability, air and moisture stability, high polarity, the ability to dissolve inorganic or organic compounds including some polymers, amphiphilicity, surface and interfacial properties, as well as a varied range of solubility and miscibility with water and other solvents. Moreover, ionic liquids additionally present other desirable properties, such as good ionic conductivity, wide electrochemical window [16,17,18] and high flash point [19].

Therefore, the structure of the ionic compound can be designed so that it exhibits the desired physical and chemical properties. Properly planned synthesis using uniquely selected types of ions results in obtaining an ionic liquid characterized by optimal properties for strictly defined needs. New structures of ionic liquids along with new methods of their synthesis and areas of their practical industrial applications are constantly being sought for.

**Figure 1:**
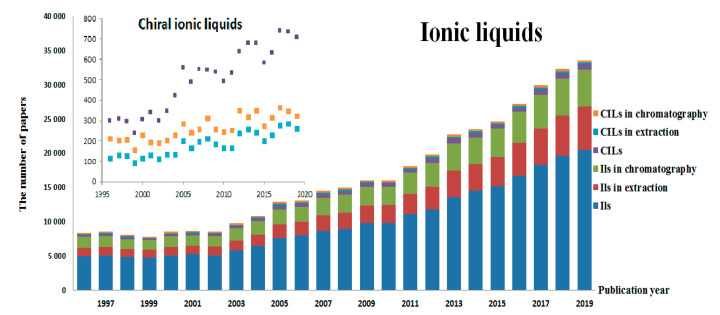
Number of published scientific papers on ionic liquids (ILs) and chiral ionic liquids (CILs) and their applicability in the field of extraction and chromatography depending on the year of their publication (1996–2019), based on the Science Direct database.

Over 20 years of research on these compounds has resulted in an impressive number of publications, which may be illustrated by the fact that in the Scopus database the phrase ionic liquids (searched in: titles articles, abstracts and keywords; until April 2020) is highlighted in over 93 thousand scientific papers. At the same time, an interest in replacing the organic solvents as a principal resource of environmental contamination with nonvolatile ionic liquids has largely evolved (Figure 1).

More recently, chiral ionic liquids (CILs) have been recognized simultaneously as nonmolecular solvents as well as chiral agents having potential of chiral discrimination capabilities and high specificity towards targeted enantiomers.

This increasing importance is due to the specific structural character of the CILs group, where either cationic or anionic part or both parts can be chiral, which makes them enantioselective.

Based on the strength of the enantioselective power of either cation or anion, high stability, reusability and efficiency, chiral ionic liquids have spread in many fields of analytical chemistry. The use of chiral ionic liquids in extraction and chromatography has increased in the last decade, as can be seen in Figure 1 (inset), although not as fast as in the general class of ionic liquids. The growing trend of interest in CILs points to the existing potential of these compounds for future considerations. There are many review papers concerning the utility of ionic liquids in analytical separations [20,21]. To date, only a few reviews on the synthesis and utility of chiral ionic liquids have been published. Some of them focus their attention on the synthesis of CILs [22,23], the role of CILs in chiral recognition of the enantiomers using various analytical techniques such as NMR and spectroscopy [24,25], liquid and gas chromatography [24,25,26] and capillary electrophoresis [24,26].

This review collectively describes the advantages of using CILs for enantioseparation in tandem with extraction including aqueous biphasic system (ABS) extraction systems, solid–liquid two-phase systems, liquid–liquid extraction systems with hydrophilic CILs, liquid–liquid extraction systems with hydrophobic CILs, solid-phase extraction and induced-precipitation techniques and separation techniques such as liquid chromatography and countercurrent chromatography.

## 2. Classification and Application of ILs

**Figure 2:**
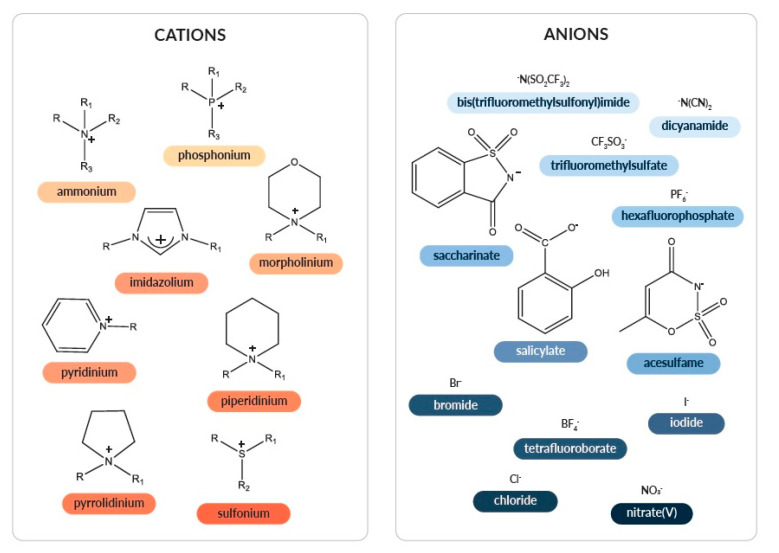
Diagram of the division of ionic liquids due to the type of cation and anion. R, R_1_, R_2_, R_3_—mainly alkyl, aryl, hydroxyl groups and hydrogen atoms.

In the literature, the classification of ionic liquids is fairly diverse and flexible; with the following criteria to be considered: chemical structures of both cation and anion constituents, physical state or physicochemical properties. Figure 2 presents a diagram of the division of ionic liquids regarding the type of cation and anion.

The most common cationic parts include imidazolium (im, e.g., C_1_C_2_im, emim, EMIM: 1-ethyl-3-methylimidazolium), ammonium (N_x x x x_, e.g., N_1 8 8 8_: methyl(trioctyl)ammonium), pyridinium (py, e.g., C_6_py: 1-hexylpyridinium), phosphonium (P_x x x_, e.g., P_4 4 4 12_: tributyl(dodecyl)phosphonium), sulfonium (S_x x x_), pyrrolidinium (pyrr), piperidinium (pip), morpholinium (morph), etc. The anionic part includes: chloride (Cl), bromide (Br), iodide (I), tetrafluoroborate (BF_4_), hexafluorophosphate (PF_6_), nitrate(V) (NO_3_), dicyanamide (DCA, N(CN)_2_), bis(trifluoromethylsulfonyl)imide (NTf_2_), trifluoromethylsulfate (TfO), acesulfame (Ace), saccharinate (Sacc), salicylate (Sal), etc.

A large combination of these salts (10^18^ ionic liquids are theoretically possible) can be designed [27,28]. By incorporating relevant ions into the structure-specific compounds, suitable functionality can be achieved. Almost limitless structural possibilities of cation and anion combinations mean that the design of ionic liquids for certain applications creates limitless possibilities.

The obvious consequence of the rapidly expanding interest in ionic liquids is the growing number of applications in which these ionic salts are being used [10,29,30,31], for purposes as diverse as catalysis [32,33], fuel cells [34], safe electrolytes [35,36], lubrication [37,38], biomass treatment [39] and energy storage [17,40,41]. In the field of analytical chemistry, ILs have gained greatest popularity in various types of extractions, separations and identification techniques, i.e., gas chromatography (GC), liquid chromatography (LC), capillary electrophoresis (CE), electrochemistry or mass spectrometry (MS) [24,26,42,43]. The first attempts to use ionic liquids as solvents for kinetics or electrochemistry were described by Swain in 1967 [44]. Ionic liquids, as solvents, have gained popularity in various industries also due to the reduction of costs through the possibility of their recovery and efficient reuse.

Increasing the diversity of ionic liquid structures resulted in the adoption of new rules for labelling them, with reference to the special (dedicated) functional group that characterizes them, to the original features of a given salt—or regarding their use, e.g., as sweet [45], energetic [46], chiral [47], fluorescent [48], polymeric [49], magnetic [50] and many others. On the other hand, the possible methods of the structural combination of ionic liquids focusing on expanding their structure with specific components resulted in the introduction of another term, namely task-specific ionic liquids (TSILs). It should be added that the association of ionic salts with the TSILs group is done by the introduction of a special functional group to the cation and/or anion, which provides ionic liquids with special properties and/or reactivity [51].

Another interesting group of ionic liquids are chiral ionic liquids, whose distinguishing feature is the possession of, at least, a single-point chiral center. This category includes the salt with a stereogenic atom/atoms in the positive part, the negative part—or both ions of the molecule, simultaneously [22,52]. Mostly used precursors are derived from the “chiral pool”, which is an efficient, economic and simple way of obtaining enantiomerically pure ILs with central chirality. Among the optically active ionic liquids, salts with axial [52,53] or planar [52,54] chirality as well as chiral spirals having a character of ionic liquids should be mentioned [55]. It is worth pointing out that most of the chiral ionic liquids contain one or more functional groups, hence—their membership in the group of “functionalized chiral ionic liquids” (FCILs) [56,57]. As this functional group is intended to perform a targeted task, these functionalized ionic liquids may also be tagged as task-specific ionic liquids.

## 3. Chiral Ionic Liquids (CILs)

The first example of chiral quaternary salts, which should be considered as a prelude to the creation of a new group of ionic liquids, commonly called chiral ionic liquids, were N-heterocyclic carbenes of imidazole, synthesized by Herrmann et al. in 1996 [58]. However, at that time these chiral imidazolium chlorides were not yet referred to as CILs. The name “chiral ionic liquids” was used for the first time by Howarth et al. [6] in 1997, who described stable chiral dialkyl imidazolium bromide, as Lewis acid—applied as the catalyst in the Diels–Alder reaction, offering high enantiomeric purity of the product. The chiral anions, for example, amino acids, lactic acid, borates, camphorsulfonates, can also occur as components of ionic liquids. In 1999, Seddon et al. [7] synthesized 1-butyl-3-methylimidazolium lactate (BMIm)([Lac) (Table 1), known to be the first reported CIL with a chiral anion. CILs containing chiral anion were obtained by an anion-exchange process occurring between 1-methyl-3-butylimidazolium chloride and *S*-2-hydroxypropionate. However, Seddon’s research has shown that the mere presence of a chiral cation or anion does not provide a chiral distinction. To guarantee enatioseparation, the chiral ionic liquid must have an appropriate structure with a sufficient number of sites for binding to the enantiomer. Therefore, to ensure multiple interaction sites, in recent years, many methods for the synthesis of chiral ionic liquids containing amino acids rich in various functional groups such as hydroxyl, amino, thiol, thioether, carboxyl and amide [59,60,61,62,63] were widely described.

**Figure 3:**
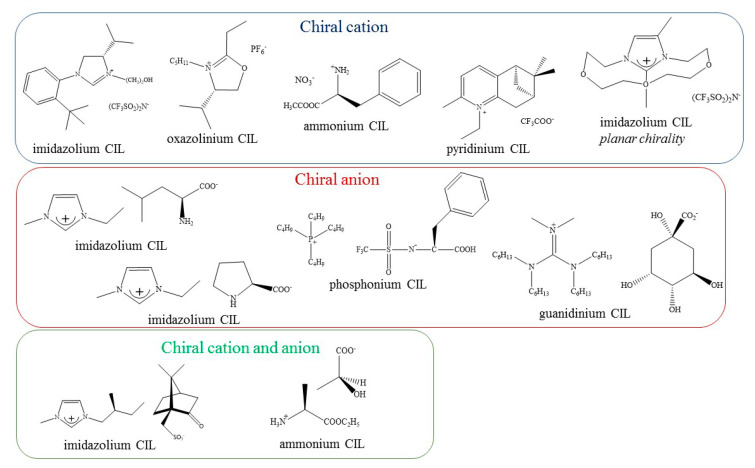
Classification of CILs.

Since then, many other CILs have been proposed [22,64,65], which were derived from carbohydrates, amino acids, alkaloids and others. Certainly, one of the most impressive aspects worth highlighting is the successful use of CILs in various applications (Figure 3, Table 1), mainly in: asymmetric synthesis, spectroscopy, chromatography, extraction, membrane separation, electrochemistry, liquid crystals and in biotechnological areas.

Raw materials, like sugars, amino acids, nicotine, menthol, etc.—which are environmentally sustainable and renewable—Often make up the center of the chirality of CILs. CILs can be used in a number of ways in biotechnology (examples are given in Table 1). The compounds presented may be successfully applied as microbial agents [66,67,68], wood protection agents [69] and plant resistance inducers [68]. Their biologic effectiveness often exceeds that of the known quaternary ammonium salts, [66,67,68,69]. The implementation of naturally occurring substituents significantly improves the biologic activity of the salts tested.

Several chiral ionic liquids, which have been synthesized, have either a chiral cation, a chiral anion or both. An example of which is shown in a work written by Yu et al. [79], describing the chiral recognition of newly synthesized CILs containing cations with an imidazolium group and anions based on a borate ion with spiral structure and chiral substituents. The authors studied the possibility of enantiomeric recognition of chiral constituents of ILs toward both enantiomeric ions and other chiral molecules such as a quinine derivative by NMR including ^1^H{^15^ N} heteronuclear single quantum coherence (HSQC). The results have shown that synthesized CILs exhibit significantly higher enantiomeric recognition capacity concerning quinine in a solvent with lower dielectric constants (CDCl_3_ compared to CD_3_CN) at higher CILs concentration. Additionally, a stronger chiral recognition was found for an anion with a larger substituent group (e.g., phenylmethyl showed a greater chiral recognition compared to the phenyl group). The authors recommended the obtained chiral ionic liquids for various applications, including the role of chiral solvents.

## 4. CILs in Analytical Chemistry

Optically pure enantiomers are used commonly as food additives, drugs or substrates for chemical synthesis. Their isolation and chiral recognition process is based mainly on high-performance liquid chromatography, membrane separation, extraction or electrochemical sensors. However, these methods have several drawbacks, such as a relatively low separation selectivity, expensive chiral column, high consumption of organic solvents and complicated analytical procedures. Therefore, to overcome these disadvantages, there exists a dire need of establishing a rapid, low-cost, efficient, environmentally friendly procedures for the resolution of racemic mixtures.

### 4.1. CILs in Enantioselective Extraction

Asymmetric synthesis allows direct creation of a pure enantiomer [80]. However, this is a labor-intensive method requiring the use of expensive raw materials or specific enantioselective catalysts [81,82]. A much cheaper alternative is the synthesis of racemates, followed by enantioseparation [83,84]. The most commonly used enantioseparation techniques are crystallization and chromatography [83]. Each of them, however, has limitations such as the ability to form conglomerates and the need for enantiomeric enrichment. Chromatography, on the other hand, suffers from the high cost of chiral columns and the limited capacity to carry out the process on a larger scale [83,85]. Due to this, the enantioselective liquid–liquid extraction (ELLE) techniques are becoming increasingly popular, which ensures low costs, as well as high simplicity and a great performance speed [84]. To ensure enantioselectivity, ELLE requires the presence of a so-called chiral selector. The formation of “chiral selector–enantiomer” complexes [86] thanks to intermolecular interactions (“three-point model”) is the basis of the final chiral recognition. The best-researched ELLE systems contain organic solvents and chiral selectors derived from β-cyclodextrin or tartaric acid [87,88,89,90,91,92]. However, nowadays, as recommended by green chemistry and sustainability [93,94], the use of volatile organic solvents should be limited. Water two-phase systems (ABS) provide such conditions as they do not generate pollution in the environment [95,96]. The use of ABS for the resolution of racemates involves two separate strategies. The first is based on the addition of a chiral selector, i.e., β-cyclodextrin derivatives, copper-β-cyclodextrin complexes, tartaric acid derivatives, proteins or microbial cells to ABS formed from a polymer–polymer combination, micellar systems of polar solvents and salts [97,98,99,100,101,102,103,104,105,106,107,108,109,110,111,112,113]. The second method utilizes the addition of one substance that acts simultaneously as a phase former and a chiral selector [114,115,116]. Recently, chiral ionic liquids (CILs) have found application as chiral phase formators as well as chiral selectors in ABS [8]. There are only a few studies on CILs use in ABS for enantioseparation, despite having many advantages, i.e., they can provide chiral cationic and/or anionic groups [22], they can act simultaneously as chiral selectors and solvents. However, this is not a homogeneous solvent, because ionic liquids are highly organized structures that can be described as polymeric supramolecules connected by hydrogen bonds. After mixing with other molecules, they become nanostructured materials with regions of varied polarity [117].

#### 4.1.1. Partitioning Systems Based on Chiral Ionic Liquids

Recent advances in the developments of ILs include looking for new TSILs to expand their usefulness in the enantioselective separation. Chiral task-specific ionic liquids could act as both solvents and one of the substrates with functional groups, specifically as hydrogen-bond donors in ion-pair creation being a sensor to chiral recognition [118,119,120,121,122].

Abraham et al. [123] have proven that both hydrophobic and hydrophilic ionic liquids are suitable for the creation of partitioning systems. A mixture of water and hydrophobic ionic liquid may be regarded as a liquid–liquid extracting system, whereas hydrophilic ionic liquid and kosmotropic salt solution as ionic liquid-based ABS.

Aqueous biphasic system (ABS) is the technique suitable for the separation and purification of different compounds, mainly drugs and biomolecules such as proteins and antibodies, enzymes, cells and other biologic samples [124]. This extracting system was already used in the eighties of the previous century [125,126]. Typically, ABS is created by mixing solutions of two different polymers such as polyethylene glycols or dextrans or a polymer and salt solutions at a certain temperature. After mixing, two aqueous immiscible phases are created. Gutowski et al. [127] showed for the first time that by the addition of kosmotropic salts (K_3_PO_4_) to a hydrophilic IL (1-butyl-3-methylimidazolium chloride), similar kind of partitioning system may be formed.

The two immiscible phases are created by mixing hydrophobic ionic liquids with aqueous solutions. During liquid–liquid extraction, vigorous agitation with the aim to increase contact between phases, ensuring increase of the mass transfer and accelerating the kinetics of the extraction process is required. In this case, the ionic liquid phase is located at the bottom. As a result of the salting-out process from aqueous solution by kosmotropic salts addition, the position of the IL phase can change into a top one [128,129,130]. The upper phase containing a concentrated solution of IL and an extracted analyte can be directly injected into the chromatographic columns.

**Figure 4:**
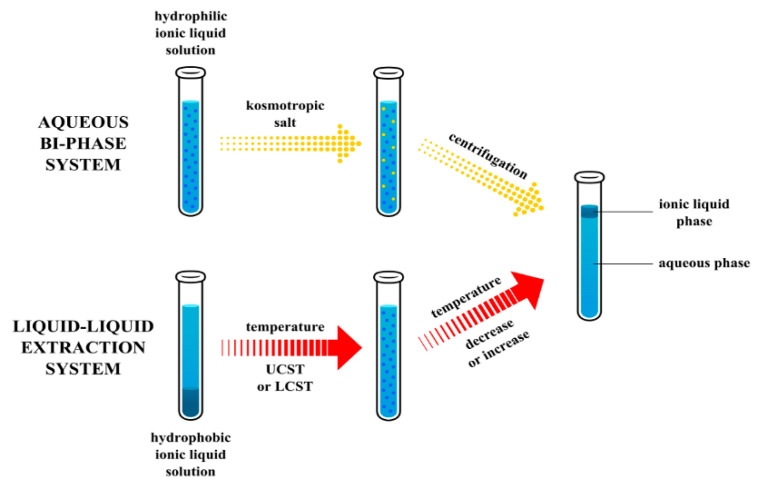
Mechanism of phase separation for hydrophobic and hydrophilic ionic liquids.

To solve the inconveniences of a two-phase system containing hydrophobic ionic liquids, we can take advantage of the dependence of solubility on temperature change. Since the miscibility of ionic liquids (ILs) with water is temperature-dependent, ILs can form one homogeneous phase from the hydrophobic IL and water at certain critical solution temperature (an upper critical solution temperature-UCST or a lower critical solution temperature—LCST). A homogeneous mixture can be converted again into a two-phase system by changing the temperature. This separation technique based on thermomorphic behavior of ILs is known as homogeneous liquid−liquid extraction (HLLE), temperature-induced phase separation (TIPS), coalescence extraction or phase-transition extraction (PTE). The scheme of phase separations for hydrophilic ionic liquids and ionic liquids exhibiting thermomorphic behavior with UCST and LCST is presented in Figure 4.

#### 4.1.2. Aqueous Biphasic Systems (ABSs)

More recently, some trends of polymers and organic solvents replacement by ionic liquids in the formation of two-phase systems have been observed [131,132,133,134,135,136,137,138,139,140]. There exist several studies devoted to the evaluation of the salting-out ability of the inorganic salts and a broad variety of hydrophilic ionic liquids through the formation of ABSs [141,142,143,144,145]. After mixing of the components of this extraction system, phase separation appears very fast, but it sometimes requires a centrifuge to be separated. This simple technique is suitable for the extraction/isolation/preconcentration of either ionic or neutral compounds [146,147,148,149,150,151].

It should be emphasized that most of the researchers agree that liquid−liquid demixing and inducing the formation of a two-phase system is an entropy-driven process and could be explained based on ΔG_hydr._ (the Gibbs free energy of hydration) of the salt ions [139,140,141,142]. The ability of conventional electrolytes to induce the ionic liquid-based ABSs follows the Hofmeister series dividing the compounds into salting-out and salting-in species depending on the degree of hydration [152]. According to this series, assessing the ability of the cations and the anions to salting-out of proteins, ions are classified as kosmotropic (highly hydrated) and the chaotropic (weakly hydrated) ones:

citrates^3−^ > SO_4_^2−^ > HPO_4_^2−^ > F^−^ > Cl^−^ > Br^−^ > I^−^ > NO_3_^−^ > ClO_4_^−^ > N(CH_3_)_4_^+^ NH_4_^+^ > Cs^+^ > Rb^+^ > K^+^ > Na^+^ > H^+^ > Ca^2+^ > Mg^2+^ > Al^3+^

highly hydrated
weakly hydrated

To define the conditions necessary to create the two-phase systems by mixing hydrophilic ILs with various salts, phase diagrams described by Merchuk et al. of the empirical model are usually utilized [153]. The binodal curves are determined experimentally through the method which is known as “cloud point titration”.

**Figure 5:**
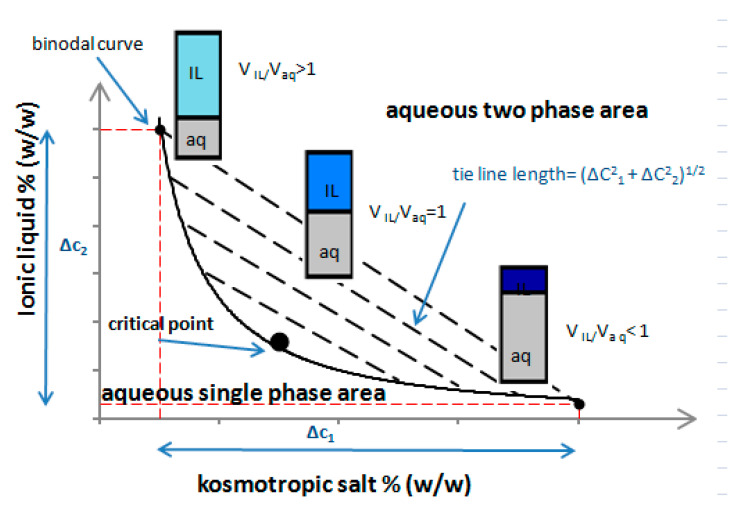
Illustration of the phase diagram. The bottom-phase compound (kosmotropic salt % *w*/*w*) is plotted on the abscissa and the top-phase compound (IL % *w*/*w*) is plotted on the ordinate. Three exemplary vials represent the volume ratios of ionic liquid top phase (V_IL_) to aqueous bottom phase (V_aq_) situated on the same tie-line; the difference in concentration of kosmotropic salt and ionic liquid between the two phases is represented by (∆c_1_) and (∆c_2_) respectively; the critical point of the binodal curve can be determined by connecting the midpoints of series of the tie-lines (------).

Figure 5 shows an exemplary phase diagram. The binodal line of the phase diagram illustrates concentrations of both the ionic liquid and salt at which phase separation is possible. By connecting any two points located on the binodal line, quantitative compositions of the two separated phases could be estimated. The stronger ability to induce the formation of ABS is visible as the larger the two-phase region situated above the binodal curve.

Each point on the tie line illustrates different combinations of components in the two-phase system, but the key matter to improve the extraction efficiency, which was also confirmed by other authors, is the correct choice of not only the kind of ILs and the salting-out species, but also the right proportion of each other [154,155]. It is the ratio of phases V_IL_/V_aq_ that will affect the pre-concentration factor. Moving along, the tie–line coordinates denote systems with different volume ratios. In addition, a critical point is present on the binodal at which the composition and volume of both phases are equal. According to the graphical illustration presented in Figure 5, the phase volume ratio of IL-phase to aqueous phase gradually decreased with either the increasing mass of the kosmotropic salt or the amount of water. This seems to be reasonable, considering the higher hydrophilicity of kosmotropic salt in comparison to the ionic liquid. Hence, if the amount of an added salt increases, this causes the lowering of water content in the upper phase and the decrease of its volume. It should be stated clearly that the e.e.% value and the enantioselectivity of CIL towards isolated analyte can be achieved only by a proper design of the applied conditions, especially the ratio of CIL to salt.

Wu et al. [156] studied various ternary systems containing a new tropine type of chiral ionic liquid. The performed experiments enabled them the evaluation of the salt effect, the length of an alkyl chain in ionic liquid cation and the temperature in the formation of ionic liquid-based ABSs. The authors proved that the two-phase-forming ability decreases from the most hydrophobic (C8Tropine)pro to the most hydrophilic one (C2Tropine)pro. In turn, the kosmotropic salt that induced the strongest salting-out effect was K_3_PO_4_. This result is in agreement with the molar entropy of hydration which could be arranged in the following order:

Δ_hyd_*S* (PO_4_^3−^) > Δ_hyd_*S* (HPO_4_^2−^) > Δ_hyd_*S* (CO_3_^2−^)

Only a few papers are dealing with the separation of the racemic mixtures with ABS comprising the functional CILs [156,157,158]. In 2015, the aqueous biphasic systems based on the novel hydrophilic imidazolium-based (*R*)-CILs were introduced with the aim of the enantiomeric separation of racemic amino acids by Wu and coworkers [157].

**Figure 6:**
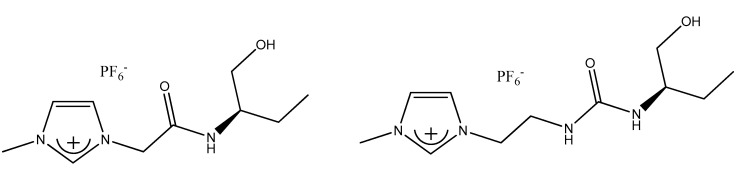
Chemical formulas of imidazolium-based (*R*)-CILs(adapted from Wu, 2015) [157].

Although two hydrophilic ionic liquids (Figure 6) were synthesized, the enantioselectivity of the first one was much better towards tested amino acids (phenylalanine, isoleucine, threonine, glutamine, tyrosine, tryptophan, serine, aspartic acid). Performed experiments proved that d-enantiomer interacted with IL, whereas l-enantiomer was driven into the top Na_2_SO_4_-rich phase.

**Figure 7:**
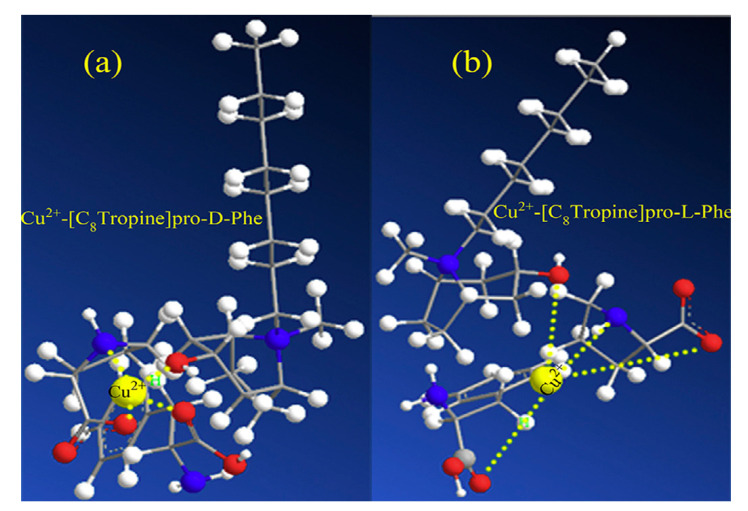
Structures and interaction of the ternary complex of Cu^2+^-[C_8_Tropine]pro-D-Phe (**a**) and Cu^2+^-[C_8_Tropine]pro-l-Phe; (**b**) yellow dotted lines represent the electrostatic interaction and the spatial electrostatic interaction of d-complex is stronger than the L-complex [156]. Reprinted from *Journal of Chromatography A*, ref. [156], Copyright (2020), with permission from Elsevier.

Later on, the “Song research group” designed a new series of chiral tropine type chiral ionic liquids with proline anion [156] differing with the length of the alkyl chain for the enantiomeric separation of racemic phenylalanine. It should be emphasized that longer alkyl chain in ionic liquid cation provides better enantioselectivity. The structures of the complexes created between chiral tropine ILs and phenylalanine enantiomers in the presence of copper ion (II) are presented in Figure 7.

Interesting alternatives to conventional ILs are Choline Amino Acid Ionic liquids (ChAAILs). They are readily biodegradable and can be prepared from renewable biomaterials without producing some toxic byproducts. A two-phase aqueous systems (ATPS) containing choline amino acid ionic liquids have been developed for the extraction of phenylalanine enantiomers [159]. Under optimal ATPS conditions 30 wt% (Ch)(l-Pro), 7.8 wt% K_3_PO_4_, 40 mmol/L HP-β-CD, 15 mg/mL phenylalanine, 4 °C operation temperature and system pH 8.0 *S*-phenylalanine was preferentially extracted into the upper phase containing the chiral selector. The separation coefficient achieved was 2.05.

Silva et al. [160] study from 2018 concerns the application of new chiral ionic liquids simultaneously as phase forming agents and chiral selectors. The authors presented ternary ABS phase diagrams composed of CILs based on quinine, l-proline and l-valine and salts (K_3_PO_4_, K_2_HPO_4_ and K_2_CO_3_) and then they examined ABS’s ability to separate the enantiomers of mandelic acid. All CILs revealed a favorably increased affinity for *S*-mandelic acid. The authors emphasized the role of electrostatic interactions between mandelic acid and CIL cations in the “three-point model” of chiral recognition. Although the CIL structure played a central role, the effect of temperature, type of kosmotropic salt, phase weight, and binding line length on the separation efficiency of enantiomers cannot be ignored.

#### 4.1.3. The Solid–Liquid Two-Phase System with CILs

The CILs with appropriate structure can interact with enantiomers in the presence of some metal ions to form complexes exhibiting different colors or solubility in a certain solvent. As this system generates the precipitate, it could be classified as a solid–liquid two-phase system. This procedure is helpful for visual chiral recognition and even for enantioseparation. So far, this approach, utilizing CILs, derived mainly from amino acids (AACILs) or amino alcohols, has been applied for the resolution of racemic amino acids [161,162,163]. In the beginning, the enantioseparation factors (np. enantiomeric excess—e.e., % or enantioselectivity—α ratio between enantiomeric partitioning coefficients) were too inefficient to be applied in practice, but more recently some improvements have been observed. In 2015, Wu et al. [157] applied ATPS with CILs to separate amino acid enantiomers achieving the maximum enantiomeric excess value (maximum e.e. %) of 53% e.e. In the same year, the authors improved this result by applying tropine-based AACILs for the resolution of racemic phenylalanine [156]. Under the optimized composition of the ABS containing 13% wt% [C_8_Tropine]pro, 13 wt% K_2_HPO_4_ and 3.5 wt% Cu(Ac)_2_, the maximum enantiomeric excess value was improved up to 65%. The above ABS systems, described in detail in the previous section, can also be classified as liquid–solid systems due to the occurrence of additional precipitation effect.

**Figure 8:**
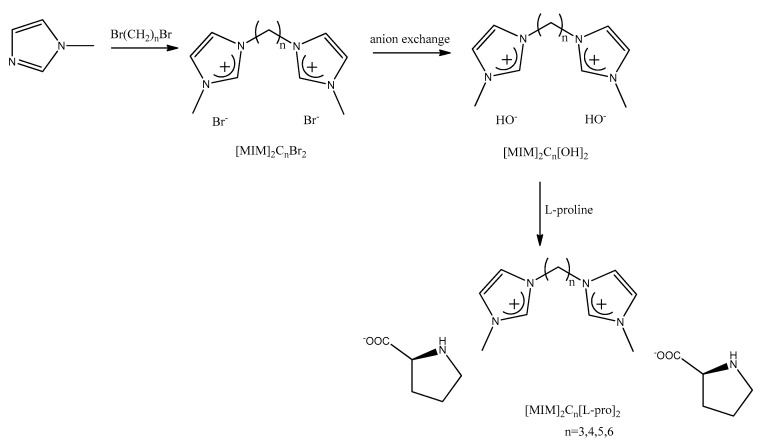
Synthesis of dication imidazolium-based chiral ionic liquids (adapted from Huang, 2017) [161].

Further improvement of the separation efficiency was obtained in a novel solid–liquid two-phase system containing dication imidazolium-based AACILs [161]. Synthesis of (MIM)_2_C_n_(l-pro)_2_ dication is presented in Figure 8. Racemic phenylalanine was used as a test analyte. The enantiomeric excess value and the yield of phenylalanine in the liquid phase containing d-enantiomer were 67.8% and 96.5%, respectively. The enantiomeric excess value and the yield of phenylalanine in the solid phase containing l-enantiomer achieved the values of 99.2% and 85.2%, respectively. The presented results indicate that the l-enantiomer interacts more strongly with chiral ILs and undergoes precipitation as the stable complex with a copper ion. Unfortunately, for other enantiomers of tryptophan, tyrosine, benzene glycine and mandelic acid, the e.e. values were much lower in the range of 28–61.5%. The authors studied additionally different factors such as the alkyl chain length of cations of ionic liquids, the amount of copper acetate, the molar ratio of ILs/Cu^2+^, the amount of water and racemic phenylalanine, the resolution time and the resolution temperature, providing the best efficiency of enantioseparation.

Ma et al. [164] proposed two functionalized chiral ionic liquids derived from (*S*)-mandelic acid (1-butyl-3-methylimidazolium mandelate and *N*-butyl-*N*-methylpyrrolidinium mandelate) beneficial for visual chiral recognition of phenylalanine, tryptophan, tyrosine and phenylglycine. In the presence of Cu(II), the resolution of racemic tryptophan achieved enantiomeric excess values (94.2% for the first CIL and 95.1% for the second one in solid phase). Using infrared spectroscopy, ultraviolet spectroscopy, thermal gravity analysis, elemental analysis and scanning electron microscope, the composition of the created complex has been specified as the following 1:1.96:0.43, representing a relation between CuSO_4_, CIL1 and D-tryptophan.

Another effective solid–liquid two-phase resolution system was proposed by Song et al. [162]. The authors developed CILs based on tropin (CnDTr((LPro)_2_ to resolution dl-phenylalanine and α-substituted carboxylic acids such as tryptophan, tyrosine, benzene glycine and mandelic acid as test substances. The best experimental conditions involving, i.e., 0.4-mol·L^−1^ of (PrDTr)(l-Pro)_2_, 0.4-mol·L^−1^ of Cu(NO_3_)_2_, 10 g·L^−1^ of dl-Phe and 5 °C), ensured the e.e. 98% value of l-phenylalanine and 99.71% of tryptophan in the solid phase. The authors claimed that the formation of l-Phe-Cu(II)-l-pro-complexes was responsible for the resolution.

An interesting phenomenon was noticed by the Wu research group which elaborated on the novel task-specific ionic liquid derivative of (*S*)-phenyl amino acid [158]. Experiments proved that while (*S*)-tryptophan treated with the new CIL in the presence of Cu^2+^ changed to a sapphire suspension, the second enantiomer remained in the solution. Moreover, after the dissolution of the whole system in DMSO, different colors of green and blue were obtained for (*R*)/(*S*)-tryptophan, respectively. These different visual responses exhibited to the enantiomers of tryptophan could be useful in the easy discrimination with the naked eye. Other racemates: (*R*/*S*)-tryptosol, (*R*/*S*)-2-phenylglycinol, and the remaining 18 amino acids were also successfully separated with the yields ranges from 90% to 94% using the same method.

#### 4.1.4. The Liquid–Liquid Extraction by Hydrophilic CILs

In 2010, Tang et al. [122] designed and synthesized novel task-specific amino acid ionic liquids (AAILs) with alkyl imidazolium cation and l-proline anion, useful for chiral separation of racemic amino acids. According to this study, new AAILs, as selectors, were coupled with Cu^2+^ ions. The formation of ternary mixed metal complexes between the AAIL ligand and the target enantiomers was responsible for the separation of racemic amino acids in the liquid–liquid extraction approach. The two-phase system was created from the ionic liquid–ethyl acetate solvents. l-enantiomers of amino acids exhibited a higher affinity to the ionic liquid phase than that of d-enantiomer. The mechanism of enantioselectivity is classified as a chiral ligand-exchange. The log *D* value obtained for l-phenylalanine was around 3.5. The authors obtained an enrichment of up to 36% e.e. of l-phenylalanine in the CIL’s phase containing 1-butyl-3-methylimidazolium l-prolinate. Ethyl acetate was used as an immiscible solvent. The authors investigated the role of the length of the CIL’s alkyl chain and found out that its elongation and reduction of solvent polarity was responsible for the increase of enantioselectivity owing to more stability of the copper complexes. The authors achieved enantiomeric excess value reaching up to 50.6% in single-stage extraction. Currently, the AAILs, either as solvents or chiral selectors, became promising tools in enantioseparation due to attractive cost and biocompatibility, as well as not complicated modification process and excellent final chiral stability.

Recently, in another relevant research [165], a CIL-based liquid–liquid extraction system based on AAILs derived from natural amino acids such as l-glutamate, l-serinate, l-phenylalanate, and imidazolium ionic liquids was developed for racemic amlodipine separation. This study describes the extraction of amlodipine in a system consisting of n-decanol and NaAc/HAc buffer solution. The aqueous phase was prepared by dissolving AAIL in buffer solution, whereas the organic phase by dissolving amlodipine into halohydrocarbon or aliphatic alcohol. The quantitative chemistry screening calculations revealed that [BMIM][Glu] has the greatest potential in the chiral recognition of amlodipine enantiomers, due to the effects of the hydrogen bridge. Under optimal extraction conditions (0.025-mol/L (BMIM)(Glu), 2.0 g/L amlodipine, pH 5.5, temperature 288.15 K) enantioselectivity of 1.38 was achieved.

In the following work [166], chiral ionic liquids, tryptophan derivatives were used, in an extraction system with an organic solvent. The maximum enantioselectivity (1.2) towards flurbiprofen enantiomers was obtained at pH 2.0 at 25 °C using 1,2-dichloroethane as an organic solvent and 1-butyl-3-methylimidazolium L-tryptophan (BMIM)(L-trp) as a chiral selector. The advantage of this work is the choice of a selector that will provide the appropriate chiral recognition capability with the help of calculations of quantum chemistry.

A CIL-organic solvent system has been frequently chosen for liquid–liquid extraction type as functional AAILs are rather hydrophilic. Immiscible organic solvents have been used for the formation of a two-phase system.

#### 4.1.5. The Liquid–Liquid Extraction (LLE) by Hydrophobic CILs

Another approach to liquid–liquid extraction of enantiomers utilizes the potential of hydrophobic ionic liquids as extraction solvents. It was proved that some hydrophobic ILs like 1-octyl-3-methylimidazolium hexafluorophosphate (OMIM)(PF_6_) appeared to be useful for transporting amino acids and amino esters [167]. Wang et al. [168] used imidazolium-based ionic liquids (C_4_MIM)(PF_6_), (C_6_MIM)(PF_6_), (C_6_MIM)(BF_4_) and (C_8_MIM)(BF_4_) as solvents for the recovery of some amino acids (l-tryptophan, l-phenylalanine, l-tyrosine, l-leucine and d-valine) from aqueous media. However, their distribution coefficient into the liquid phase [BMIM-PF_6_] is the highest only for zwitterions existing at intermediate pH s whereas cationic and anionic forms are almost undetectable. Armstrong et al. [169] measured distribution coefficient (*P*_IL/water_) values at pH close to isoelectric point (5.1) for phenylalanine, and tryptophan obtaining rather low 0.013 and 0.012 values, respectively, which were similar to the respective values of *P*_oct/water._

It should be emphasized that amino acids, as hydrophilic compounds, are difficult for conventional solvent extraction and sometimes require the addition of lipophilic agents, making them able to form extractable complexes with amino acids. The most popular complexing agents are macrocyclic crown ethers, which form hydrophobic “host–guest” type complexes with protonated amino groups of amino acids. However, even in this case, extraction into organic solvents such as chloroform is not very efficient. Alternatively applied, room temperature ionic liquids (RTIL) such as 1-butyl-3-methylimidazolium hexafluorophosphate (BmimPF_6_), containing the crown ether dicyclohexano-18-crown-6 (CE), enable efficient extraction of amino acids with varied hydrophobicity from acidic aqueous media [170]. At acidic solution, the positive form of amino acids was complexed by the crown ether and the final complex was extracted into the ionic liquid phase. The apparent distribution of these amino acids increases at low pH (1.0) after the addition of dicyclohexyl-18-crown-6 to the BMIM-PF_6_ phase.

**Figure 9:**
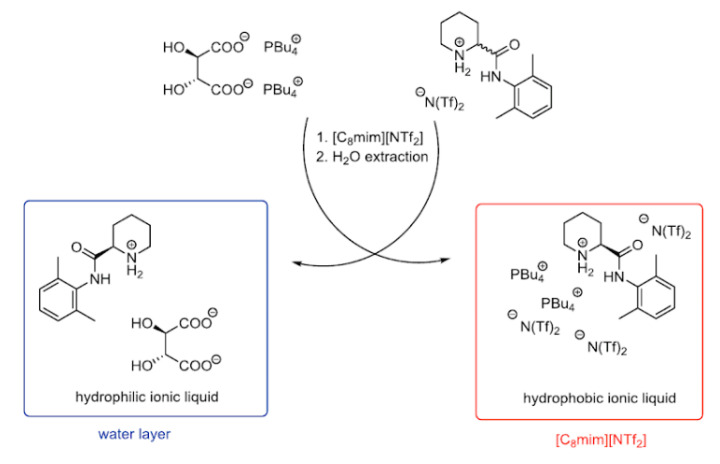
Enantioselective liquid–liquid extraction with the chiral ionic liquid [PBu_4_]_2_[*R*,*R*-tartrate] (adapted from Zgonnik, 2012) [171].

However, to achieve enantioseparation, the ionic liquid phase should be enriched with a chiral selector. In 2012, Zgonnik et al. proposed the enantioselective LLE process, without metal complexation, utilizing two different ionic liquids differing in hydrophobicity. The two-phase system was obtained by mixing highly hydrophilic CIL tetra-butyl phosphonium *R*,*R*-tartrate with hydrophobic ionic liquid 1-octyl-3-methylimidazolium bis(trifluoromethylsulfonyl)imide (BMIM)(NTf_2_) containing racemates in the cationic form. As a result of this ion switch (ion cross-metathesis) process, an enantiomeric enrichment achieved up to 30% for pipecoloxylidide at 50 °C [171]. Enantioselective liquid–liquid extraction procedure using chiral ionic liquid (PBu_4_)_2_(*R*,*R*-tartrate) is illustrated in Figure 9.

### 4.2. CILs as Stationary Phases Surface Modifiers

Solid supports can be modified by ILs including single-cation and multi-cation ionic liquids, polymeric ionic liquids as well as chiral ionic liquids. Different approaches were applied to immobilize ILs on the surface by covalent bonding or polymerization. When ionic liquids are attached to the surface of materials, they no longer constitute a true ionic liquid and can be considered rather as an ion-exchanger. These new sorbents known as ionic liquid stationary phases (ILSPs) provide a series of potential interactions such as hydrophilic, hydrophobic, hydrogen bond, π–π interaction, dipole-dipole and electrostatic interaction and found numerous applications in RPLC, normal phase (NPLC) and hydrophilic interaction liquid chromatography (HILIC). Despite so many benefits, still there are no ILSPs available commercially.

**Figure 10:**
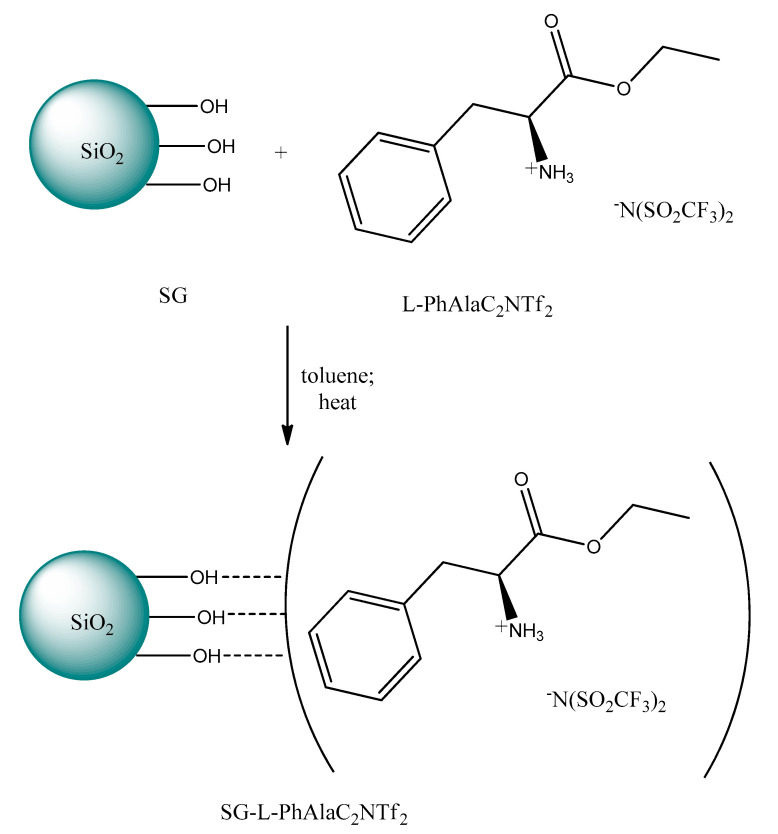
Synthetic route of the SG-l-PhAlaC_2_NTf_2_ phase (adapted from Marwani, 2014) [172].

There are only several research papers dealing with the use of CILs as the modifiers of the supporting materials. Marwani et al. [172] described the preparation of new sorbent based on the physical adsorption and hybrid combination of the chiral ionic liquid (l-PhAlaC_2_NTf_2_) and activated the silica gel (SG) surface. The selectivity of SG-l-PhAlaC_2_NTf_2_ and the adsorption capacity toward racemic mixtures of *R*-(+), *S*-(–)-1-(2-naphthyl)ethanol, d,l-tryptophan and d,l-PhAla were investigated under batch conditions. Obtained data revealed that the adsorption capacity of the new sorbent was the most enantioselective toward d-phenylalanine and equaled 97.35% at pH 3.0 after one hour of contact time. The authors proved that the adsorption of d-enantiomer of phenylalanine onto a new solid-phase followed Langmuir adsorption isotherm and pseudo-second–order kinetic models. The preparation of the SG-l-PhAlaC_2_NTf_2_ adsorbent is presented in Figure 10.

**Figure 11:**
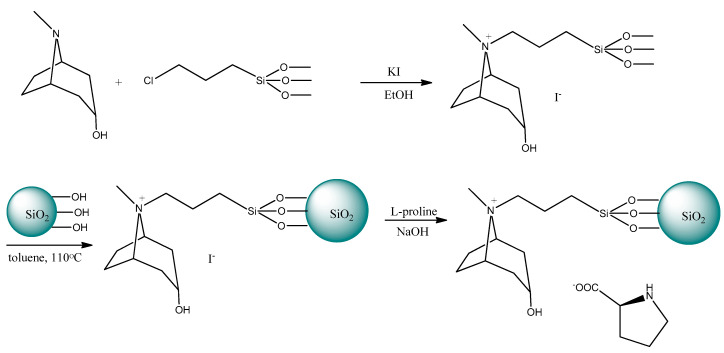
Preparation of immobilized tropine-type ionic liquid on silica gel(adapted from Qian, 2016) [173].

In 2016 Yao et al. [173] described the chemical bonding method for immobilization of the chiral tropine IL with (*S*)-proline anion on the surface of silica gel. The procedure of silica gel modification is presented in Figure 11.

Subsequently, a glass chromatographic column (height: 20 cm, i.d.: 1 cm) was packed with the ionic liquid-modified silica gel saturated with Cu^2+^ and used to separate d,l-tryptophan and d,l-phenylalanine. Due to the differences in affinity of l or d enantiomers to the chiral selector, d-enantiomers were eluted first, whereas L-ones were retained onto the functional sorbent. The authors emphasized the fact that due to smaller steric hindrance appearing in the complex of l-enantiomer with the chiral selector, it can interact more strongly with SiO_2_·Trop^+^·Pro^−^. In this research, the chromatographic process was conducted under low pressure and the elution rate was controlled at 0.1 mL/min by an air pump.

Yang et al. [174] presented a very promising feature of ionic liquids as solvents in molecular imprinting technology (MIT), which is useful in the isolation or purification of analytes on large scale. MIT uses the interactions based mainly on hydrogen bonding between the template and the monomer. However, these interactions undergo disruption in polar or protic solvents resulting in a lower imprinting efficiency and specificity in the separation of the targeted analytes. The approach proposed by Yang enhances the affinity of 4-vinyl pyridine to l-phenylalanine (l-Phe) by introducing an oil-soluble amino acid ionic liquid as a template. In this study, 1-butyl-3-methylimidazolium α-aminohydrocinnamic acid (C_4_C_1_IM-Phe) was applied to prepared (MIPs) as a template to enhance the affinity of MIP for the selective extraction of l-enantiomer of Phe from an amino acid mixture. Selective recognition of l-Phe and its isolation from the aqueous phase was possible owing to the creation of more homogeneous binding sites of MIT prepared in this process. The authors achieved the selective separation of l-Phe with a recovery of 90.6%.

In 2016 Huang et al. [175] synthesized two chiral ionic liquids (ILs), 1-ethyl-3-methylimidazole l-tartrate (EMIML-Tar) and 1-ethyl-3-methylimidazole l-lactate (EMIML-Lac), which were used for gold nanoparticles (AuNPs) modification. Modified nano-particles were applied for chiral recognition of amino acid enantiomers such as tryptophan, tyrosine and phenylalanine. It appeared that only EMIML-Tar-AuNPs was better as a chiral inducer in comparison to EMIML-Lac-AuNPs enabling satisfactory chiral recognition of tyrosine enantiomers.

In 2015, the magnetic nanospheres Fe_3_O_4_@SiO_2_ modified with chiral amino acid ionic liquid (Fe_3_O_4_@SiO_2_@HMDI-EMIMLpro) were prepared and successfully applied to separate tryptophan racemate by centrifugal chiral chromatography [176].

### 4.3. HPLC System Modified by CILs

HPLC offers direct or indirect enantioseparation methods. The indirect methods involve the addition of chiral modifiers forming diastereomeric complexes with appropriate enantiomer which is further separated by the use of conventional columns. The direct separation, on the other hand, involves the use of chiral stationary phases.

#### 4.3.1. HPLC Adsorbents Modified with CILs

Silica is considered to be the most frequently used stationary phase matrix in high-performance liquid chromatography (HPLC) due to the possibility of linking different materials to its particles. In 1985 Moreira and Gushikem [177] used, for the first time, the silica gel functionalized with 3-(1-imidazolyl) propyl groups to pre-concentrate metal ions from ethanol solutions. However, only at the beginning of the 21st century, the modification of a stationary phase by CILs and their utilization for HPLC was described by Liu et al. [178]. This new stationary phase prepared by the immobilization of IL 1-(mercaptopropyl) alkyl-3-hexylimidazolium tetrafluoroborate onto the silica support, was applied to separate series of ephedrines utilizing a mobile phase containing low amounts of organic modifier (1% methanol) in phosphate buffer at pH 3.0. Since that time, chromatographic columns modified with ILs have attracted increasing attention mainly owing to their interesting selectivity and relatively low free-silanol activity.

This trend has been continued and developed by many researchers describing different methods with the aim to attach various IL cations/anions to chromatographic sorbents [179,180,181,182,183,184,185,186,187,188,189,190,191,192,193]. Most authors focused their attention on understanding the role of modified phases in the retention process. They pointed out to multi-modal retention mechanism involving the contribution of multiple interactions like hydrophobic, electrostatic and hydrogen bonding between the confined IL and on the surface and separated solutes. Unfortunately, there are only a few instances of applications of CILs as CSPs in HPLC. CSPs can be prepared using different synthetic approaches, providing bonding of CILs on the surface of spherical porous silica and hence constituting the so-called surface-confined ionic liquids (SCILs).

**Figure 12:**
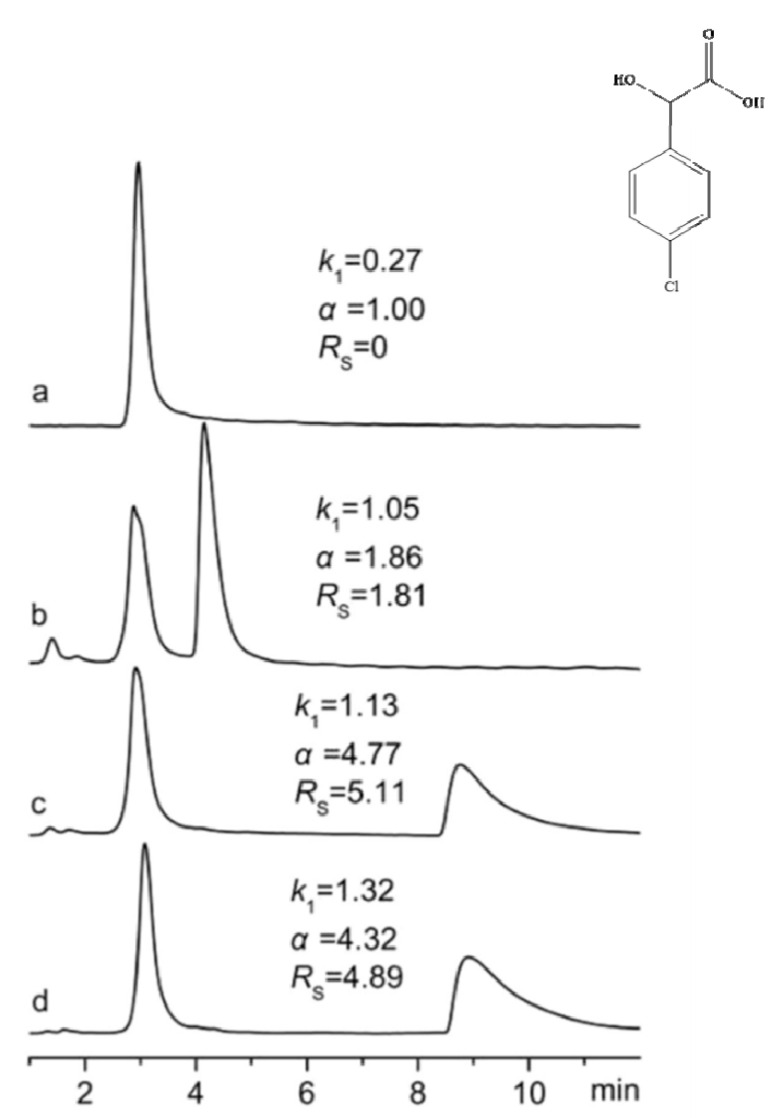
Effect of the mobile phase composition on enantioseparation of 4-chloromandelic acid resolved by CSP 1. Mobile phase: (a) ACN/(water/TFA) (60/40, *v*/*v*); (b) ACN/(water/TFA) (70/30, *v*/*v*); (c) ACN/(water/TFA) (80/20, *v*/*v*); (d) ACN/(water/TFA)(90/10, *v*/*v*), flow-rate: 1.0-mL·min ^−1^, UV: 254 nm [194]. Reprinted from *Analytica Chimica Acta*, ref. [194], Copyright (2020), with permission from Elsevier.

In 2016, Wang et al. [194] synthesized novel chiral stationary phases (CSPs) by bonding chiral imidazolium derivatives of the amines: 1-phenylethylamine and 1-(1-naphthyl)ethylamine on the surface of silica gel. Obtained CSPs appear to be valuable to separate racemates of mandelic acid and its derivatives, 1-phenylethylamine derivatives, 1,1′-bi-2-naphthol and camphorsulfonic acid. It was proved that the acetonitrile amount in mobile phases was crucial for enantiorecognition (Figure 12).

**Figure 13:**
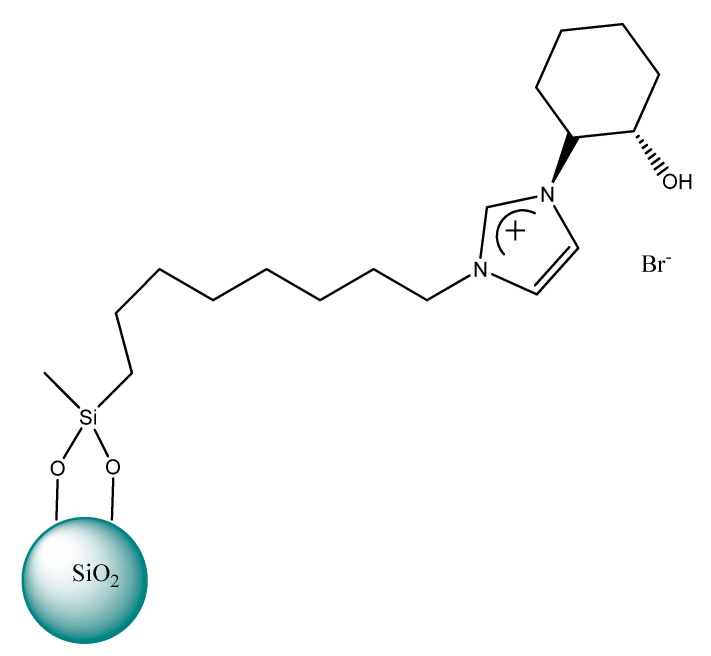
Chiral surface confined ionic liquid stationary phase (adapted from Kodali, 2014) [195].

In 2014, Kodali and Stalcup [195] synthesized the chiral selector 2-(1*H*-imidazol-1-yl)cyclohexanol, which was used to modify the silica (Figure 13). The chiral separation of the enantiomers of phenanthro [3,4-c] phenanthrene (hexahelicene) was achieved under normal phase conditions using hexane/dichloromethane (10:90) mobile phase. Although baseline resolution was not achieved, the resolution value was 0.91 and the selectivity ratio was only 1.07, it should be emphasized that the enantioseparation of hexahelicene still poses a challenge to researchers.

More recently, novel CSPs, based on chiral imidazole bonded to the surface of the silica sphere with strong anion exchange properties, were prepared by He et al. [196]. The ionic liquid chiral selector was synthesized by ring-opening of cyclohexene oxide with imidazole or 5,6-dimethylbenzimidazole and modified by appropriate substitute groups. The obtained CSPs exhibited excellent enantioseparation towards acidic racemates. The retention was governed by the ion exchange process as well as the steric hindrance, π-π and hydrogen bonding interactions. The chromatographic results proved that the chiral recognition ability was affected mainly by the kind of substituent on the chiral selector.

##### HPLC Stationary Phases Modified with CILs in Tandem with Cyclodextrins

Another approach is represented by the modification of common chiral selectors by ionic liquids to enhance enantioseparation and then bonded to silica particles. Cyclodextrins are commonly used in chiral HPLC stationary phases, here are some advantages to using derivatized ILs in tandem with cyclodextrins. Since Armstrong et al. [197] discovered cyclodextrins (CDs) as the new chiral selectors, the CD-based chiral stationary phases (CSPs) have been widely applied in enantioseparation based on their unique cavity structures. More recently, imidazolium or 1,2,3-triazolium substituted cyclodextrins have been covalently bonded onto silica gels. Obtained novel cationic β-CD CSPs exhibited enantioselectivity towards both polar and nonpolar compounds [198,199,200].

Wang et al. in 2008 [198], prepared a chiral ILSP by grafting imidazolium with cyclodextrins and then bonding them to silica particles. The cationic moiety on β-CD derivative interacting with analytes through hydrogen-bonding and electrostatic forces was important for the separation of the racemates. However, the length of the alkyl chain of the imidazolium moiety proved to be crucial in the enhancement of chiral recognition, affecting the strength of interaction with the analyte. The longer octyl chain of the imidazolium, the moiety gave much better resolution of racemic α-phenyl alcohols as test analytes in comparison to CSPs bearing a methyl group. Furthermore, phenylcarbamoyl groups appeared to be more beneficial over 3,5-dimethylphenylcarbamoyl groups on the cyclodextrin ring [199]. Scientific group of Ng and coworkers [201,202] prepared the whole series of CSPs modified by cationic β-CD derivatives for the separation of aryl alcohols, flavanone, thiazide and amino acid derivatives.

**Figure 14:**
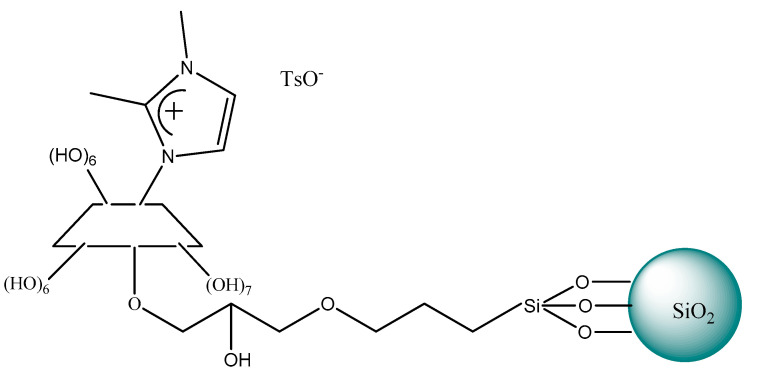
Functionalized ionic liquid-bonded chiral stationary phases: mono-6-deoxy-6-(1,2-dimethylimidazolium)-β-cyclodextrin tosylate CSP (adapted from Zhou, 2010) [198].

Since then, cyclodextrins (CDs) linked to ILs started to be used as chiral selectors for HPLC enantioseparation. The stationary phase prepared by grafting ILs with cyclodextrins and then bonded to silica particles was described a few years later in 2010 by Zhou et al. [198,203]. Novel stationary phases were based on silica-bonded cyclodextrins (CDs) that were further functionalized with 1,2-dimethylimidazolium or 1-amino-1,2,3-triazolium cations and variable anions nitrate or tosylate (Figure 14). The obtained phases were very useful to separate a series of eighteen racemates including nitroalcohols, hydroxylamines, alcohols, as well as two racemic drugs. Satisfactory enantioselectivity with a resolution of over 1.5 was obtained on these columns and acetonitrile-based polar mobile phases for almost all the racemates studied. The authors emphasized the role of the counter anion of CIL and electrostatic attraction from the confined cationic moieties contributing to the chiral recognition process. It was noticed that both the cationic moieties of imidazolium ion and triazolium ion interact differently with the analytes. It appeared that imidazolium ion exhibited stronger H-bonding interactions in comparison with triazolium ion. Zhou et al. [198] proved that CSPs modified with a chiral selector containing nitrate anion ensured the better resolution of the acidic racemic drugs analytes and chiral aromatic alcohol derivatives, due to weaker basic properties than tosylate and smaller steric hindrance participating ion exchange process.

Furthermore, an important role in the interactions between the analytes and the chiral selectors is played by the ionic strength of the acetonitrile based polar-organic mobile phase as well as the structure of analytes specifically the position of the substituent on the aromatic ring.

**Figure 15:**
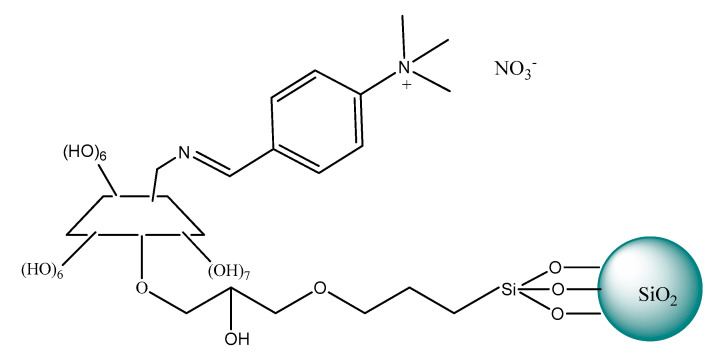
Structure of mono(6-deoxy-imino)-beta-cyclodextrins chiral stationary phases (adapted from Li, 2014) [204].

**Figure 16:**
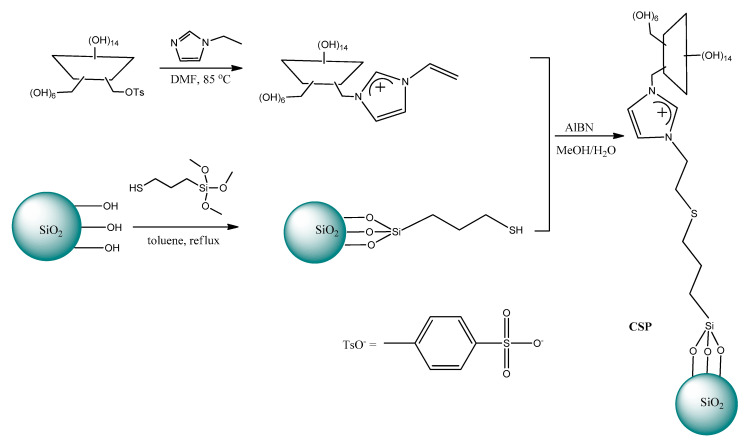
Synthetic pathway of the novel cationic chiral stationary phases (CSPs) (adapted from Yao, 2014) [205].

In other studies, the use of cyclodextrins modified with ionic liquids to obtain chiral adsorbents was continued. Four new benzimido-β-CDs-cyclodextrins functionalized by ionic liquids (Figure 15) were prepared and linked to silica gel [204]. These chiral stationary phases (CSPs) were applied in high-performance liquid chromatography (HPLC) to the resolution of enantiomers of 1-phenyl-2-nitroethanol derivatives, aromatic alcohols and ferrocene derivatives. The authors achieved excellent enantioseparations emphasizing the cooperation of both ionic substituents on β-CD derivatives and ion-pairing interactions as the main factors contributed to the separations. More beneficial, for efficiency of enantioseparation, appeared to be those CILs with a smaller volume of a cation. This is because of better steric fitting between CILs and the analytes creating inclusion complexes. The tosylate anions responsible for hydrogen-bonding and π–π interactions, have exhibited favorable separation of the compounds with smaller molecular volume whereas nitrate anions were more useful for the compounds with larger volumes. In 2014, Yao et al. [205] demonstrated a novel cationic native cyclodextrin (CD) chiral stationary phase (CSP) prepared by thiol-ene click chemistry. Authors anchored vinyl imidazolium β-CD onto thiol silica and applied it to enantioseparation of dansyl (Dns) amino acids, carboxylic aryl compounds, flavonoids, Tröger’s base and voriconazole. The obtained CSP exhibited a very good resolving ability with α > 1.1, R_s_ > 1.5 towards acidic chiral compounds. Obtained CD-CSP based on an imidazole linkage appeared to be more efficient in comparison to another CD-CSP based on a triazole one. The synthetic pathway of the novel CSP is presented in Figure 16.

Rahim et al. prepared new β-cyclodextrin functionalized by imidazolium ionic liquid and further immobilized onto silica gel to the resolution of several racemic mixtures of β-blockers (propranolol, metoprolol, pindolol and atenolol). The authors achieved the complete separation of enantiomers of two racemates, namely propranolol and metoprolol through the formation of inclusion complexes and π-π interactions of enantiomers with the modified stationary phase (β-CD-BIMOTs) [206].

Beside imidazolium ionic liquids, the β-CD can be derivatized also by quaternary ammonium linked to the cyclodextrin cavity and used as chiral solid support [201,204]. Owing to this modification, more than 20 analytes such as aromatic alcohols and ferrocene derivatives were separated with the resolution factors in the range of 0.86–4.95.

A recent work by Berthod et al. [207] has described the preparation of novel ILs functionalized cyclofructan 6 (CF6) macrocyclic-oligosaccharides and their bonding to silica gel to be used in HPLC as stationary phases. The cyclofructans were derivatized by propyl imidazole, methyl benzimidazole, dimethyl aminopropyl, pyridine and dimethyl aminophenyl. The triethoxysilyl-propylisocyanate was used as a linker to bond the derivatized cyclofructans to 5 µm spherical silica gel particles. Almost 80% of the studied chiral compounds were resolved in the normal-phase system (NP) applying heptane/ethanol mobile phase, for around 10% polar organic mode (PO) with acetonitrile/methanol eluent system appeared the most beneficial whereas the reverse phase (RP) system with eluent containing acetonitrile/pH 4 buffer was completely insufficient.

#### 4.3.2. HPLC Mobile Phase Modified with ILs

The reversed-phase high-performance liquid chromatography (RP-HPLC) on a C18 column is the most common technique applied in order to separate the bioactive compounds. However, RP-HPLC of the basic solutes causes some kind of disadvantage such as severe peak tailing or low system efficiency. These changes are commonly described as “silanol effects”. These effects are mainly due to the interaction of the basic compounds with free silanols on a silica support. Many methods have been developed for eliminating silanophilic interactions using mainly enrichment of the eluent with surfactants or amines [208]. Nahum and Horváth [209] explained the role of these silanol masking agents in decreasing the retention of charged analytes and improvements in the peak symmetry. Amine additives are also added to mobile phases to separate basic enantiomers on polysaccharide-based chiral stationary phases [210,211]. However, the addition of amines gives improvements in peak shape rather due to kinetic effects than to silanol interactions. Moreover, a ‘‘memory effect”, changing the column performance, was observed with columns that had previously been exposed to amine additives. The n-butylamine memory effect was demonstrated by Ye et al. [210] on CHIRALPAK AD. Stringham studied a memory effect for diethylamine (DEA), on three different polysaccharide chiral stationary phases [211]. This study included a large group of amine-containing analytes. A beneficial memory effect of DEA was observed in hexane-containing mobile phases, whereas in the case of the polar eluent system, this effect disappeared.

Ionic liquids have also been added with success to the mobile phase with the aim of suppressing silanophilic interactions in RP-HPLC [212]. ILs applied as the mobile phase additives can improve peak symmetry and calibration curve linearity. It should be emphasized, however, that ILs dissolved in a molecular liquid lose their unique ionic liquid properties and become ordinary salts. The critical limitation of the HPLC technique is the maximum pressure allowed (<15 MPa/30 cm), which excludes the use of pure ionic liquids as solvents in HPLC. ILs are too inherently viscous liquid, due to the strong interactions occurring between cations and anions, to be used as main solvents. If the viscous liquid were applied as the eluents of HPLC, the column would have been exposed to too high pressure, resulting in damaging the polymer matrices of the columns. Even the least viscous IL still has 20 times much higher viscosity than that of water. For instance, a viscosity of 1-allyl-3-methylimidazolium chloride, a very commonly used IL in a liquid state at room temperature is over 2000 cP. Therefore, pure ILs are not applicable as eluents of HPLC. The problem of high viscosity disappears after mixing with organic solvents such as methanol or acetonitrile. In recent years, ionic liquids have been used as additives for mobile phases in RP-HPLC. There are many examples of successful separations with unique elution patterns of amines, acids and biomaterials, proving the usefulness of ILs in HPLC. Even a small, millimolar amount of ionic liquids greatly improved the shape of the peaks, mainly basic substances such as aromatic amines, β-blockers, catecholamines and alkaloids [213,214,215,216,217,218,219]. It should be emphasized that most of the ionic liquids used as additives in the mobile phases contained an imidazole cation (EMIM, BMIM, HMIM, OMIM) associated with a relatively large anion (Br, Cl, PF_6_, BF_4_). Due to the poor hydration of both components, they can be classified as chaotropic ions, which are capable of creating ion-pairing interactions. One should also realize that ionic liquids are compounds that have a double nature. Accordingly, both charged ions may exhibit synergistic or antagonistic effects on the retention process. Furthermore, because ions can exhibit different hydrophobicity, they can be sorbed onto stationary RPLC phases as well as interact specifically with analytes in the mobile phase. This issue has been extensively studied by Gritti and Guiochon [220,221,222,223,224,225].

##### CILs Added to the Achiral Chromatographic System

CILs can play various roles to improve resolutions in liquid chromatography. Apart from the use of CILs as stationary phases modifiers, they can also be added to the mobile-phase as chiral additives. CILs, as mobile phase additives, play a role of the so-called chiral selectors (CSs), chiral ligands. It should be emphasized that owing to the dual nature of ILs, their cations, as well as anions, are able to influence the chromatographic process. Furthermore, the ions can adsorb onto the stationary phase. This sorption process depends on the lyotropy of ions and provides the creation of unique interphase.

There are only a few papers describing chiral ionic liquids application as additives to the eluent. The first application of CILs in liquid chromatography was described in 2006 by Yuan et al. [226]. The authors described the potential of 10-mmol/L (*R*)-*N*,*N*,*N*-trimethyl-2-aminobutanol bis(trifluoromethylsulfonyl)imide ionic liquids as chiral selectors in the mobile phase consisting of H_2_O and CH_3_CN to resolve eight analytes using a commercially available C18 ODS column.

**Figure 17:**
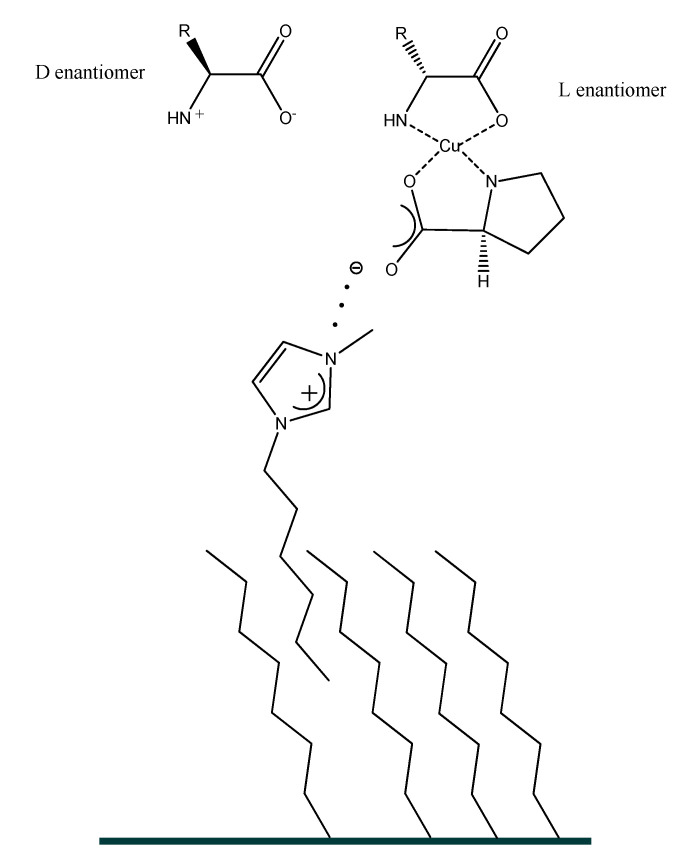
Retention mechanisms of (Cu(AAIL) (l-amino acid) complexes on the high-performance liquid chromatography (HPLC) column. Dots between l-Pro and alkylimidazolium cations represent formation of ion pairs (adapted from Liu, 2009) [227].

Liu et al. [227] pioneered the application of amino acids derived ionic liquids (AAILs) in enantioseparation of amino acid enantiomers basing on the chiral ligand exchange mechanism in 2009. Four 1-alkyl-3-methylimidazolium C_n_MIM-l-proline (l-Pro) with different alkyl chain lengths coupled with Cu^2+^ were used as chiral mobile-phase additives. The chiral recognition was based on the diverse behavior of the diastereomeric complexes formed between CILs and enantiomers of phenylalanine, histidine, tryptophan, tyrosine used as test racemates. The authors applied fluorescence detection mode with an ex/em wavelength (λ) 215 nm/295 nm. The mobile phase contained only 1 mM of chiral mobile phase additive, 0.5 mM Cu(Ac)_2_ and MeOH (15% *v*/*v*) in water providing very good resolution on C18 column. Baseline separation was achieved for racemic phenylalanine. The 1-phenyl-1,2-ethanediol racemate was resolved with selectivity factor α value of 3.86. The obtained enantioselectivity was affected by the AAIL cation. It was noticed that the longer the alkyl chain in imidazolium head group from C_2_ to C_8_ was associated with a better resolution. The authors suggested that these excellent separations are related to the ion-pairing process occurring between the analyte and imidazolium cation of the chiral selector undergoing sorption on the surface of the stationary phase owing to the strong interactions of its long-chain with the hydrophobic C18 surface. The scheme showing the hypothetical retention mechanisms is presented in Figure 17.

A few years later, similar experiments were carried out by Yang et al. [228]. As chiral ligands, the derivatives of l-proline ILs coupled with Cu(II) were used to separate the tryptophan enantiomers. The application of the optimum conditions enabled to obtain a successful separation of the enantiomers with a resolution value of 2.30 and a selectivity factor of 1.25. The effects of the cation (the l-proline derivatives) and anion (NO_3_^−^, SO_4_^2−^, CF_3_COO^−^) on enantioselectivity were also investigated by Qing et al. [229]. The best experimental conditions were the following: 8-mM Cu(OAc)_2_, 4-mM (l-Pro)(CF_3_COO), 20% *v*/*v* MeOH. However, the resolution value was smaller (1.89) in comparison with the previous investigation performed by Yang et al. [228].

Other amino acid-derived chiral ionic liquids with CuSO_4_·5H_2_O as mobile-phase modifiers were used in ligand exchange chromatography in 2011 [230]. Bi et al. used them to ofloxacin enantiomeric separation. The authors examined the effects of the kind of ligand (based on alanine, valine, phenylalanine and leucine), the concentration of Cu(II), organic modifier, pH of the mobile phase and temperature. AAILs were used as a ligand (4.0-mmol·L^−1^) together with CuSO_4_·5H_2_O (3.0-mmol·L^−1^) in methanol/water (20/80, *v*/*v*), as the optimal mobile phase for ligand-exchange chromatography. When chiral amino acid-based ILs were used as additives, the authors observed that generally, the enantioselectivity increased with increasing alkyl chain length of the amino acid anions which was the highest for (C_4_MIM)(Leu). In turn, the resolution decreased with increasing alkyl chain length of the IL cations and it was the smallest for (C_8_MIM)(Leu). It was quite a different trend in comparison to that one reported by Liu et al. All reasons for that can be explained by weaker electrostatic interactions of ILs with longer alkyl chain with Cu(II) and a steric hindrance causing an increase in repulsive interaction.

##### CILs Added to the Chiral Chromatographic System

In numerous cases, satisfactory enantioseparation cannot be achieved using a single chiral selector. Therefore, double systems were used to improve enantioselectivity. Much better resolution of enantiomers was obtained for the first time in capillary electrophoresis (CE) by using several chiral selectors simultaneously, based on the synergistic effect of such combined systems [231,232,233,234,235,236]. To date, there are only a limited number of reports on the application of CILs as chiral selectors forming synergistic systems in HPLC.

Feder-Kubis et al. [237] described, for the first time, a synergistic system basing on the chiral glycopeptide stationary phase (Chirobiotic) with CILs containing bicyclic monoterpene moiety as mobile phase additives in HPLC. The above system was successfully used for the resolution of enantiomers of mandelic acid, vanillylmandelic and phenyllactic acid. CILs derivatives of (1*S*)-*endo*-(–)-borneol and (1*R*)-*endo*-(+)-fenchol were used as chiral selectors. It was proven that the addition of the chiral ionic liquids and elongation of the alkyl group of CILs significantly affect the increasing differences in the binding energy of enantiomers to teicoplanin.

Later on, Flieger et al. [238] studied the specific cooperative effect of a semisynthetic glycopeptide antibiotic and chiral ionic liquids containing the (1*R*,2*S*,5*R*)-(–)-menthol moiety on the chiral recognition of acidic enantiomers. Experiments were carried out on an Astec Chirobiotic T chiral column applying the mobile phase with the addition of not only CILs, but also chaotropic salts. The thermodynamic measurements and the docking simulations revealed that the steric adjustment between cyclohexane ring of CIL and the β-d-glucosamine ring of teicoplanin together with hydrophobic interactions between the decanoic chain of teicoplanin and the alkyl group of the tested analytes were responsible for the chiral recognition.

### 4.4. Counter-Current Chromatography Modified with CILs

Counter-current chromatography (CCC), in which a biphasic system (the mobile and stationary phase) is composed of two immiscible liquids, can be considered as an advantageous alternative for all chiral separations’ techniques described above. This is mainly because of the high loading capacity and low solvent consumption. However, to achieve enantioseparation, the choice of the proper chiral selector appears to be significant.

Because ILs can form biphasic liquid systems; they can be potentially applied in CCC. Unfortunately, the first trials failed mainly due to their high viscosity. To solve this problem, in 2017, a new separation methodology with specially designed instrument with a large internal bore and limiting pressure build-up, was designed [239].

Despite the fact that the CILs have become common chiral selectors (CSs), chiral ligands or background electrolyte (BGE) additives in many separations techniques, only one work describes the applications of CILs in high-speed counter-current chromatography (HSCCC) [240]. Wang et al. developed the combination of amino acid ionic liquids (AAILs) namely Cu(II)-[1-butyl-3-methylimidazolium][l-Pro] and hydroxypropyl-β-cyclodextrin (HP-β-CD) to the enantioseparation of intractable naringenin (NRG) racemic mixtures by a high-speed counter-current chromatography (HSCCC). HSCCC system has been optimized taking into account the consumption of chiral selectors, pH and temperature. Ninety-eight percent purity of isolated enantiomers was achieved. From 10 mg of the natural racemic compound, 4.5 mg (+)-NRG and 4.1 mg (–)-NRG were obtained. The mechanism of chiral recognition was studied using UV-Vis and NMR spectra. The synergistic effect of the current chiral selectors, i.e., Cu(II)-[BMIM][l-Pro] and HP-β-CD was associated with the difference in thermodynamic stability of quaternary Cu(II), [BMIM][l-Pro], HP-β-CD and NRG complexes. Basing on the above achievement, AAILs appear to be very promising additives for chiral CCC, as single chiral ligands as well as synergistic components of any chiral system.

## 5. Conclusions

Over the past decade, an enormous progress was achieved in the design and synthesis of chiral ionic liquids including, as well as in the search for their potential applications. The use of renewable materials for many of those optically active salts synthesis appears to be extremely important from both economic and ecological sides.

There are many diverse applications of chiral ionic liquids, mainly in fields of chemistry, but also in physics and biologic sciences and those results encourage further research. Aspects of applications in various areas of analytical chemistry are particularly highlighted in most of the chiral ionic liquids’ reviews. The scientific papers on those specific salts often reveal not only interesting uses, but also important issues for potential industrial implementations. Over the last years, ILs have been used as an “environment-friendly” alternative to organic industrial solvents. Due to the fact that they are not a source of harmful waste products, they meet the requirements of so-called “green chemistry”.

It should be emphasized that chiral recognition is a particularly important area for the pharmaceutical industry regarding the varied pharmacological activity and the different pharmacokinetic profile of the drug enantiomers in the human body. According to the Food and Drug Administration regulation of 1992 [241], single enantiomers must be the leading component of currently approved drugs. That is why quantification of enantiomeric purity is required. In enantioseparation or enantio-purification, the selection of suitable chiral selectors and solvents appears to be a key matter for the efficiency of these processes. CILs-based extraction and CILs-based chromatography provide a solution to the above- mentioned needs simultaneously.

The use of CILs can bring about higher enantio-extraction yields and enantio-purification factors when we compare them with conventional methods.

This review is a compilation containing a description of structures’ diversity, classification, properties of CILs and their usefulness for the enantioseparation process in different extraction methods and chromatography in the last years. In conclusion, CILs appear to be promising solvents with additional chiral functionality. The obtained enantioseparation coefficients (e.g., enantiomeric excess—e.e.% or enantioselectivity—α ratio between enantiomeric partitioning coefficients) have improved significantly in recent years as a result of systematic research. However, the design of new task-specific ionic liquids with suitable physicochemical properties and improved efficiency as the chiral solvents or chiral inducers is still a great challenge providing novel opportunities in further research. Since most separation processes provide the opportunity to recycle and reuse components of the system, this would allow separation on an industrial scale.

## Figures and Tables

**Table 1:** Selected chiral ionic liquids with various applications.

Structure	Application	Reference
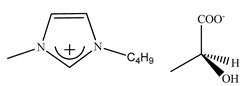 chiral: (*S*)-2-hydroxypropionate derivative; (l-lactate)	Diels–Alder reaction	[7]
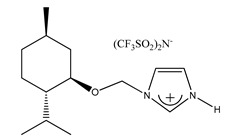 (1*R*,2*S*,5*R*)-(–)-menthol derivative	Diels–Alder reaction	[33]
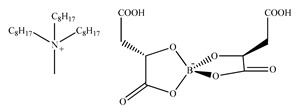 l-(–)-malic acid derivative	Aza–Baylis–Hillman reaction	[61]
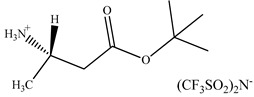 l-alanine; d-alanine derivatives	chiral recognition in spectroscopy: ^19^F NMR, fluorescence	[70]
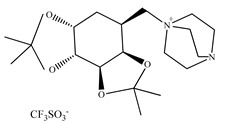 d-galactose derivative	chiral recognition in ^19^F NMR; organocatalyst in the enantioselective reduction of aromatic prochiral ketones	[71]
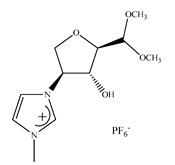 d-xylose	chiral recognition in ^19^F NMR	[72]
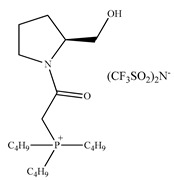 *S*-prolinol derivative	chiral recognition in ^19^F NMR	[73]
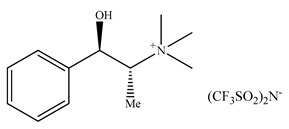 (1*R*,2*R*)-(–)-pseudoephedrine derivative	chiral phase for chromatography	[74]
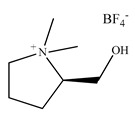 l-proline derivative	enantiomeric separation in capillary electrophoresis	[75]
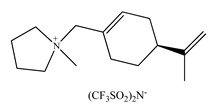 (*S*)-perillyl alcohol derivative	electrochemical enantiodiscrimination	[76]
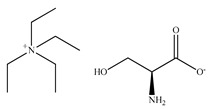 l-serine derivative	electrochemistry—high ionic conductivity	[77]
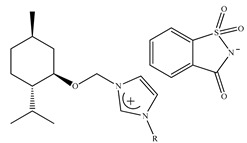 R = CH_3_ ÷ C_12_H_25_ (1*R*,2*S*,5*R*)-(–)-menthol derivative	liquid crystal (R = C_9_H_19_); wood protection agents (R = C_6_H_13_ ÷ C_12_H_25_)	[69,78]
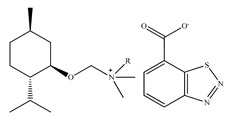 R = C_9_H_19_; C_12_H_25_ (1*R*,2*S*,5*R*)-(–)-menthol derivative	microbial agents; plant resistance inducers	[68]
